# Supplementary figures and images for: Intercellular forces driving stratification in a two-layer corneal epithelium: Insight from a Voronoi cell-based simulation model
Source: PLoS Comput Biol. 2026 Feb 20;22(2):e1013279. doi: 10.1371/journal.pcbi.1013279 (PMC12935309; doi:10.1371/journal.pcbi.1013279)

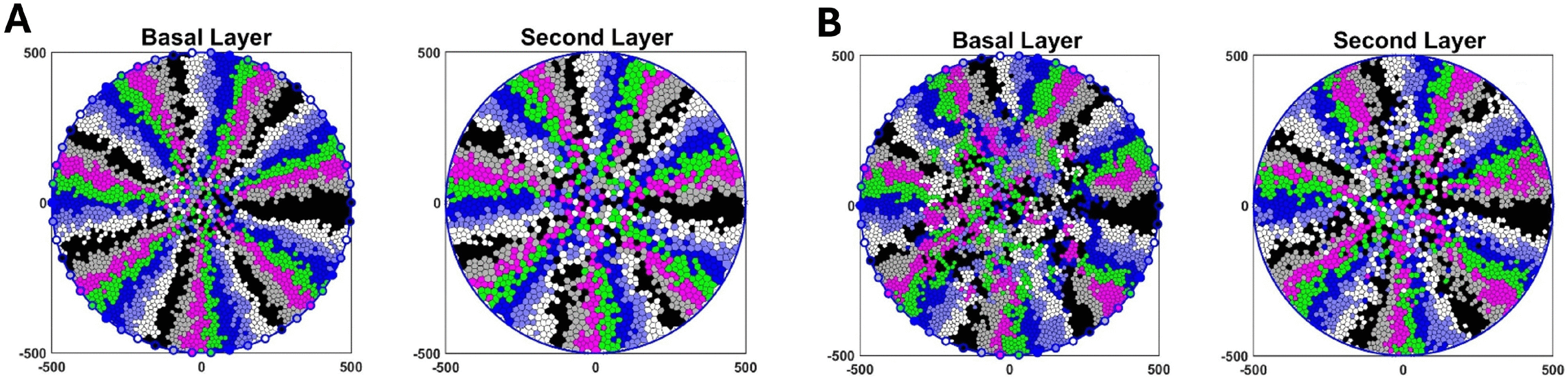

Supplement: S2 Fig — Simulation of the centripetal growth in the basal and the second layer for (A) nmax = 1, (B) nmax = 5 to see how the centripetal pattern changes for the extreme values. (TIFF) [file pcbi.1013279.s002.tiff]

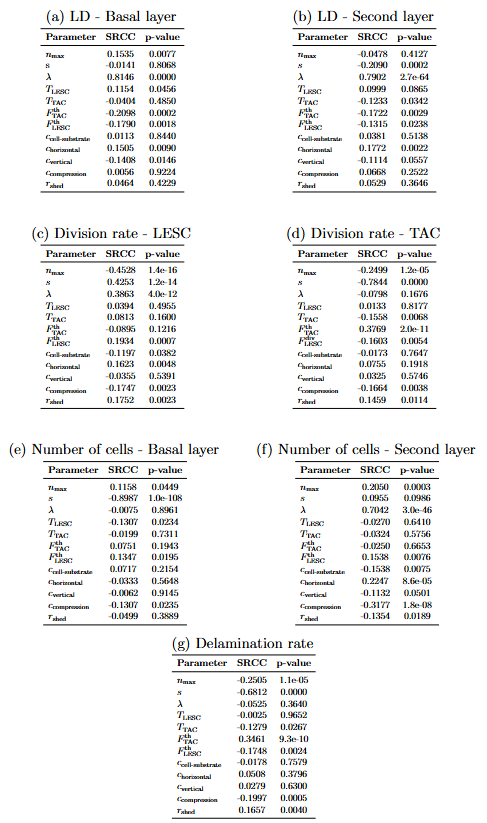

Supplement: S1 Table — shows SRCC and p-values between model parameters and (a) LD - Basal layer, (b) LD - second layer, (c) Division rate - LESC, (d) division rate - TAC, (e) Number of cells - Basal layer, (f) Number of cells - Second layer and (g) delamination rate. (TIFF) [file pcbi.1013279.s004.tiff]
