# Supplementary material for: Intercellular forces driving stratification in a two-layer corneal epithelium: Insight from a Voronoi cell-based simulation model
Source: PLoS Comput Biol. 2026 Feb 20;22(2):e1013279. doi: 10.1371/journal.pcbi.1013279 (PMC12935309; doi:10.1371/journal.pcbi.1013279)
Supplement: S1 Appendix — The structure of this ODD is based on the framework proposed by Grimm et al. [50] for describing agent-based model. (PDF) [file pcbi.1013279.s005.pdf]

# ODD protocol of the mathematical model

The structure of this ODD is based on the framework proposed by Grimm *et al.* [1] for describing agent-based model.

## 1 Purpose

The model is used to investigate the key mechanisms of the basal and second layers of the corneal epithelium. Specifically, it can be employed to determine the cell division rate in the basal layer, the delamination rate from the basal layer to the second layer, as well as to investigate cell-substrate adhesion, cell-cell forces, and the centripetal growth pattern in both layers.

## 2 Entities, state variables, and scales

- The corneal epithelium is stratified with 6-8 layers. At the moment, the model considers two layers: the basal layer and one layer of wing cells (second layer).
- The basal and the second layer are modeled as flat circles with radius 500 $\mu$m.
- We consider three cell types:
  - Limbal epithelial stem cells (LESCs) on the limbus, or outer rim, of the basal layer.
  - Transit amplifying cells (TACs).
  - Terminally differentiated cells (TDCs).

  Each cell has the following attributes, or state variables:

  1. Position: Each cell is characterized with its x and y coordinates in a Voronoi diagram.
  2. Lineage identification: Each stem cell is assigned a unique lineage identification code, which is inherited by all of its progeny within the cornea, ensuring that cells originating from the same stem cell share the same lineage identifier.
  3. Type: Each cell is characterized by a type (LESC, TAC or TDC).
  4. Cell cycle clock: Time elapsed since last cell division is saved as a cell cycle clock for each cell.
  5. Proliferation. Cell division only happens in the basal layer, based on the cell type.
     (a) LESCs divide asymmetrically to produce an LESC and a TAC in the basal layer. The resulting LESC stays in the limbus and the resulting TAC is placed a slight distance of 0.05 $\mu$m from the parent LESC in a random direction towards the interior of the cornea.

(b) We assume TACs divide symmetrically to a maximum number of divisions $n_{\max}$. After division of a parent TAC, the number of divisions of two daughter TACs increases by 1. When a TAC's number of divisions reach $n_{\max}$, it becomes a TDC and no longer divides.

(c) Cell division rate is obtained by taking the cumulative average of the ratio of dividing cells over total number cells with the capacity to divide in each time-step.

6. Death: We assume LESCs, TACs and TDCs death rate in either layer is zero. In fact, TACs primarily turnover by moving upward through the delamination process and then are shed from the surface of the cornea [2].

7. Movement: Centripetal and vertical migration from the basal layer to the second layer are the two main orientations for epithelial cell movement.

# 3 Process overview and scheduling

The following operations are conducted for all cells:

1. Death: Determine which cells die. If a cell dies, mark it as "to die". In the model, we assume death rate of all cells is zero.

2. Delamination: For cells in the basal layer that did not die, determine which cells delaminate using the criterion in Section 3.3. If a cell delaminates, mark it as "to delaminate".

3. Division: For cells in the basal layer that did not die or delaminate, determine which cells divide using the criterion in Section 3.2. If a cell divides, mark it as "to divide".

4. Update system: Remove cells marked as "to die". Move cells marked as "to delaminate" from the basal layer to the second layer. For cells marked as "to divide", generate a new cell using the algorithm outlined in Section 2 proliferation part.

5. Horizontal movement: For all cells in both layers, use the cell movement submodel in Section 3.1 to determine the displacement vectors of every cell. Then, update all cell positions at the same time. The movement step is iterated five times per time step. This approach allows the spring forces to approximately reach equilibrium before other biological processes, such as cell division or delamination, are applied.

# 4 Design concepts

1. Basic principles: We make the following assumptions:

   - The cornea is in steady state and cells are motile.
   - The cell lineage progression is LESC, TAC and TDC.
   - There is 50 LESCs on the rim.
   - Cells feel pressure from its neighbours in their own layer. Basal cells feel pushing or pulling force from the cells above them in the second layer.

2. Emergence: Centripetal movement and cell division and delamination rates are emergent phenomena driven by a cellular force network.

3. Adaptation: Cell movement depends on the spring forces obtained from Hooke's Law. They replication is density dependent describe by $F^{\text{neigh}}$ in Section 3.2.

4. Objectives: The cells do not pursue or aim to achieve any specific objectives; rather, their behaviors emerge from local interactions and predefined rules governing cellular dynamics.

5. Learning: Individual cells do not change their adaptive traits over time in response to experience.

6. Prediction: there are no adaptive traits in the model, so the agents in the model do not make any predictions.

7. Sensing: cells can sense the pressure exerted by their neighbors in their own layer. Basal cells can sense the pulling or pushing force from the cells in the second layer.

8. Interaction: There is cell-substrate adhesion and cell-cell force between cells in their own layers and within layers.

9. Stochasticity: There is no source of stochasticity in the model.

10. Collectives: There is no pre-determined collective behavior in the model.

11. Observation: Position, lineage identification, type and cell cycle clock are recorded for all cells in the model at every time step.

# 5 Initialization

To generate our initial population, we begin with a rim of LESCs around the basal layer and no other cells in the basal or second layers. We run the simulation until it reaches a steady state, which we found to occur by time 5000 hours. We use these steady-state configurations as our initial populations for our simulations.

# 6 Input data

There are no external input data that influence the model during the simulations.

# 7 Submodels

At each time step, the model is updated according to three processes: cell movement, cell division and cell delamination, mentioned in Sections 3.1, 3.2 and 3.3.

# References

[1] Grimm V, Railsback SF, Vincenot CE, Berger U, Gallagher C, Deangelis DL, et al. The ODD protocol for describing agent-based and other simulation models: A second update to improve clarity, replication, and structural realism. JASSS. 2020;23(2).

[2] West JD, Ã NJ, Collinson JM. Evaluating alternative stem cell hypotheses for adult corneal epithelial maintenance. World J Stem Cells. 2015;7(2):281-99.
