# Supplementary material for: Intercellular forces driving stratification in a two-layer corneal epithelium: Insight from a Voronoi cell-based simulation model
Source: PLoS Comput Biol. 2026 Feb 20;22(2):e1013279. doi: 10.1371/journal.pcbi.1013279 (PMC12935309; doi:10.1371/journal.pcbi.1013279)
Supplement: Attachment — Submitted filename: Response to Reviewers.pdf [file pcbi.1013279.s006.pdf]

Neda Khodabakhsh Joniani, PhD Candidate

School of Mathematics and Statistics

The University of Sydney

Re: Response to Reviewers

Dear Editor and Reviewers,

We would like to sincerely thank you for your valuable time, constructive comments, and insightful suggestions on our manuscript entitled "Intercellular forces driving stratification in a two-layer corneal epithelium: Insight from a Voronoi Cell-Based simulation model", co-authored with Dr. David Martinez Martin, Prof. Peter Kim and A\Prof. Guy Lyons. We greatly appreciate the effort you put into carefully reviewing our work, which has helped us to improve the quality and clarity of the paper.

We have carefully considered all comments and revised the manuscript accordingly. Detailed responses to each reviewer's points are provided below, with explanations of the changes made or justifications where appropriate. All modifications in the revised manuscript are clearly indicated using blue text for ease of review.

We hope that the revised version addresses all concerns and meets the expectations of the reviewers and the editor.

Thank you once again for your thoughtful and helpful feedback.

Sincerely,

Neda Khodabakhsh Joniani
(on behalf of all authors)

School of Mathematics and Statistics Faculty of Science Room 807 Carslaw Building F07 The University of Sydney NSW 2006 Australia T: +61406699728 E: n.khodabakhsh@sydney.edu.au

Reviewer #1

Major Questions.

1. What happens if there is some damage in the base layer? Would be the system adaptive enough to generate the similar patterns even in response to these perturbed environment?

This is a great question, and we are looking into recovery from damage to the basal layer and other kinds of wound healing in a subsequent work. A summary of our findings is below; however, presenting it as part of this work would require extensive articulation of the problem and results, which would compromise the clarity, focus, and scope of the current paper. We consider that such results will be better place in a separate work, as that would allow us to provide the depth and rigor the topic deserves, ensuring both contributions remain clear and impactful for readers.

If damage occurs in the basal layer, neighbouring cells exhibit accelerated centripetal migration toward the wound site and undergo morphological stretching to facilitate closure. This response is accompanied by an increased division rate of limbal epithelial stem cells (LESCs) and transient amplifying cells (TACs), promoting rapid basal layer repair. Typically, the basal layer is restored within 24–48 hours following injury. Once basal layer repair is complete, epithelial stratification resumes, ultimately restoring the full corneal epithelium. Consequently, the characteristic centripetal pattern is re-established across all epithelial layers.

For future work, we are extending this model to incorporate more epithelial cell layers and explicitly simulate basal layer damage, with the aim of investigating the dynamics of pattern formation and adaptive responses under perturbed conditions.

2. What is the effect of changes in c_vertical in vertical force in generating the patterns?

We thank the reviewer for this insightful question. A detailed response is provided below and has been included to the sensitivity analysis Section 5 (page 19- paragraph 7):

Vertical and horizontal forces play a crucial role under delamination conditions. We have defined linear displacement (LD) as a quantitative measure of the extent to which cell migration exhibits a centripetal pattern. Based on the sensitivity analysis presented in Section 5 (Fig. 15 (a,b)), an increase in c_vertical, can decrease LD, indicating more clonal cohesion across both layers. In contrast, a decrease in c_vertical, can increase LD and reduces the spoke-like pattern of migration.

3. In the model, the cell division occurs under two conditions. And, one of the conditions were the maximum neighbour force is less than a threshold F^{th} for LESCs and TACs. And those two threshold values are different for LESCs and TACs. How were those parameter chosen? I understand that the authors stated in Section 3.4 that two threshold values (F_LESC^th, F_TAC^th) were chosen to obtain the desired dynamics and sensitivity analysis was performed for the effect of different values. However, cell division is a critical step of the whole process and modelling. And, I wonder what are the critical dynamical changes authors expect if different set of the thresholds were chosen? Any thoughts on this?

We are grateful to the reviewer for this thoughtful question.

For the first part, we have included the following reference and corresponding calculation in Section 3.4, Parameter estimation (page 10).

Gudipaty et al. (2017) [41] reported that epithelial cells increase their division rate when cell density decreases by approximately 1.4-2.5-fold. In our model, considering a corneal diameter of 1000 μm, and an average basal cell diameter of approximately 15 μm, the basal layer contains roughly 4400 cells. A 1.4-fold reduction in cell density corresponds to an increase in cell diameter to about 17.8 μm, implying an extension of approximately 2.8 μm, from the rest length in the neighbor force (Eq. 3) for most cells. With c_neigh = 1 pN/ μm, this results in a neighbor force of F^neigh = 2.8 pN. Accordingly, we set the threshold force for TAC division to F_TAC^th = 3 pN. We assigned a slightly higher threshold F_LESC^th to promote sufficient TAC production within a shorter time period. Interestingly, despite the higher F_LESC^th compared to that of TACs, the model exhibits an emergent behaviour in which the division rate of LESCs remains lower than that of TACs.

Interestingly, despite the higher F_LESC^th compared to that of TACs, the model exhibits an emergent behaviour in which the division rate of LESCs remains lower than that of TACs. Regarding clonal cohesion, increasing both LESC and TAC threshold values leads to a more organized, spoke-like pattern (i.e. smaller LD values, Fig. 15a, b).

For the second part, we have addressed this point and clarified our explanation in the fifth paragraph on page 19.

An increase in F_LESC^th promotes more frequent LESC division, thereby increasing its division rate, while increasing F_LESC^th decreases TAC division rate, indicating that TACs divide more slowly. In contrast, there is a negative correlation between F_TAC^th and the LESC division rate, suggesting that an increase in TAC division rate reduces the necessity for LESCs to divide (Fig.15(c)). Moreover, while increasing F_TAC^th enhances the TAC division rate, increasing F_LESC^th has the opposite effect, resulting in a reduced TAC division rate (Fig. 15(d)).

4. It would be great to see time courses of spatial distributions of cells from different lineages and patterns in both layers instead of showing the snapshot of the pattern at final time? (at t=5,000 hours). This way, we can better understand the whole dynamics.

We thank the reviewer for this important point. In supporting information, we have added the video “Clonal growth in the basal and the second layer” to see the whole dynamics.

Minor comments.

i) Page 5, 2nd line from Eq. (1): There is a typo in formula: vector F_i^V (here the location of arrow is misplaced and the notation is not consistent with vector F_i^V in Eq. (1).

We thank the reviewer for noticing this error and have corrected it.

Reviewer #2:

1. There is no specified method section in the manuscript that explain how the simulation was performed.

Thank you for this important point. We have added an ODD protocol of the mathematical model in Supplementary Information to explain how the simulation is performed.

2. Has the author tried to increase the dt = 5 to dt=8 or 10 to see the difference in the results?

Thank you for this valuable comment. This clarification has been added to the end of Section 3.4 (page 11):

We update the system with a time step dt = 5 hours. The centripetal pattern was preserved for dt <= 5 but deteriorated for larger dt. This limitation arises from the discrete-time nature of the agent-based update rules: larger time steps reduce accuracy and violate the assumptions underlying event probabilities and motion updates.

3. Also, you have taken the mouse cornea in consideration for this simulation, how would this model and simulation fit for a human eye cornea? Or has the author tried different parameters for human cornea?

Thank you for pointing this out. We have added the following explanation to the Discussion part (page 24):

This model is constructed based on parameter values derived from the mouse cornea. We expect similar qualitative behaviour in the human cornea; however, there is a lack of quantitative data for human corneal parameters. Moreover, simulating the human cornea is computationally more expensive due to its larger surface area and the likely greater number of LESCs distributed along the limbal rim.

4. Another great addition to the paper would be a time-lapse video of the simulation to see how the cells move and replace the shedding of old cells.

Thank you for this helpful suggestion. In supporting information, we have added the video “Clonal growth in the basal and the second layer” to see the whole dynamics.

Reviewer #3: Summary

Major Comments.

1. Biological Validation and Experimental Comparisons
o The model's predictions (e.g., enlarged superficial cells under high shedding, TAC buffering of LESCs) are compared qualitatively to literature observations but lack systematic quantitative validation.
o Recommendation: Integrate published data on clone velocities, cell size distributions, or division rates for at least one key parameter (e.g., centripetal velocity vs. shedding rate in Fig. 14). Explicitly state how model outputs align—or diverge—from experiments.

We thank the reviewer for this suggestion. We have added the reported division rates for LESCs and TACs alongside Fig. 16 (page 23), which shows the estimated LESCs and TACs division rates are close to the experimentally measured values.

Regarding the centripetal velocity vs. shedding rate, we have added an explanation about the published data and the model result to Section 4.4 (page 17) as follows:

In our model, we assume a shedding rate of $\mathrm{r_{shed}} = 0.0005\ /\mathrm{hour}$ [43], for which the resulting velocity is approximately 0.05 $\frac{\mu\mathrm{m}}{\mathrm{hour}}$ (see Fig. 14). This value deviates from the experimentally reported migration rate [44]. However, for $\mathrm{r_{shed}} = 0.004\ /\mathrm{hour}$, the corresponding velocity is 0.31 $\frac{\mu\mathrm{m}}{\mathrm{hour}}$, which is in the ballpark of the reported migration rate. We predict that as the layer three and the higher layers are constructed, the increase in cell size will allow for an enhancement in velocity.

2. Parameter Justification and Sensitivity
o Many parameters (e.g., force constants, thresholds, λ) are chosen to "produce desired dynamics" rather than based on measurements, risking circular logic.
o Recommendation: Provide literature-supported ranges for at least some parameters (e.g., traction forces from corneal epithelial cells). Where data are lacking, highlight model predictions as testable hypotheses rather than fixed assumptions.

We appreciate this valuable comment. We have included the following explanation in Discussion section (page 24):

Several limitations of this model should be considered. Accurately determining several parameters in our model is challenging, particularly given the difficulties to accurately access and measure physical magnitudes at the single cell level for the basal layer of stratified epithelia, including the presence of suprabasal layers and interconnectedness of cells. For this reason, certain parameters were calibrated phenomenologically to reproduce key features of epithelial behaviour.

This step is essential to test whether the model can reproduce observed biological behaviours. If the model fails to do so having parameters that can be freely adjusted, it suggests the mechanistic assumptions are incomplete or incorrect. Conversely, the fact that parameter values could be found for our model to successfully reproduce observed phenomena supports that the mechanistic principles embedded in the model are key drivers of the biological process. Further experimental progress and calibration will enable the development of more accurate and

predictive models, and a deeper assessment of the wider implications of phenomenologically tuned parameters, a limitation of this model that needs to be considered.

3. Biological Scope and Realism
o Cell death, biochemical regulation, and signalling cues are excluded, limiting relevance for real tissue behaviour.
o Recommendation: Discuss how growth factors (e.g., EGF, integrins) or apoptosis could be incorporated. At minimum, acknowledge this limitation when interpreting wound-healing dynamics.

We thank the reviewer for highlighting this point.

We have added the following explanation to the Discussion part (page 23-24):

A limitation of the current model is that it currently simulates a healthy tissue in homeostasis. In future, complexity can be added to the model to address pathological situations more realistically, such as wounding of the central cornea or limbus, genetic conditions that affect LESC lifespan and interactions with the stroma via growth factors and other effector molecules.

4. Overinterpretation of Results
o Statements such as "our model explains the presence of enlarged cells" are too strong; the model recapitulates this phenotype but does not rule out alternative mechanisms (e.g., cytoskeletal remodelling).
o Recommendation: Temper conclusions and clarify that this is a minimal mechanical model rather than a comprehensive explanation.

We agree with the reviewer that the statement overstepped what can be concluded with certainty. The following paragraph has been added to the section "Relationship of Cell Division Rates to Shedding Rate" (page 16).

It should be noted that this result does not exclude alternative biological or mechanical mechanisms (e.g., cytoskeletal remodelling, changes in cell–cell adhesion, or differential proliferation rates) that could contribute to this phenotype.

In addition, this clarification has been added to the abstract:

Interestingly, while increased shedding promotes division and delamination, excessive shedding leads to mechanical compensation through cell stretching in the upper layers. This mechanical response provides a simple, plausible explanation for the presence of enlarged cells in the superficial epithelial layers, while not excluding the potential contributions of other mechanisms.

5. nmax and Division Rate Analysis
o The focus on extreme nmax values (1 and 5) is only loosely biologically motivated since experimental estimates cluster near 2–4.
o Recommendation: Emphasize results within the physiological range; move extreme cases to supplemental data.

We thank the reviewer for pointing this out. We have now emphasized the results corresponding to the range of n_max (2–4) in the main text (Section 4.1, page 12) and the results for the non-physiological values have been retained in the supporting information section for reference.

6. Figures and Data Presentation
o Several figures (e.g., Figs. 9–11) rely on static bar plots. Emergent dynamics (spoke formation, cell size evolution) are not fully visualized.
o Recommendation: Add time-lapse visualizations, lineage trajectory plots, or size heatmaps. Overlay model predictions with experimental images where possible.

This helpful suggestion is appreciated. We have added the fluorescence micrograph of the cornea adopted from [22] to Fig. 7 (page 13) to show the model simulation, specifically the centripetal pattern, is consistent with the real image of the mouse cornea.

Moreover, we have included this explanation in the caption of Fig. 7:

"A fluorescence micrograph of a cornea from a K14CreERT2-Confetti mouse. Scale bar: 500 μm. This figure is adapted with permission from [22]."

In supporting information section, we have added the video "Clonal growth in the basal and the second layer" to see the whole dynamics.

7. Extension to Multilayer Corneal Epithelium
o The real cornea has 5–7 layers; the two-layer model captures only a subset of processes.
o Recommendation: Reframe claims as a foundational step toward multilayer modelling. Outline how additional layers could be incorporated and what new questions this would address.

We thank the reviewer for the insightful comment.

This clarification has been added at the end of the abstract:

Although the real cornea contains five to seven layers, this two-layer framework focuses on the key mechanical principles of stratification and can be viewed as a foundational step toward more comprehensive multilayer modelling.

At the end of the introduction:

This simplified two-layer model captures the fundamental mechanics of stratification and serves as a stepping stone toward multilayer representations of the cornea.

And in the discussion part (page 23-24):

Another important limitation is that the framework currently is based on two layers of the epithelium --- the basal and the second layers. To achieve a more comprehensive understanding of the corneal epithelium and its dynamics, it will be essential to extend the model by incorporating upper layers and investigating their mechanisms. We expect that the horizontal and vertical forces would be transmitted to the upper layer; however, to fully capture the stratification, an interlayer adhesive force proportional to the cell area should be introduced.

However, modelling the upper layers, particularly those that are not directly connected to the basal layer, presents a significant challenge in this approach. In this context, several questions arise, such as the relationship between the shedding rate from the uppermost layer and the cell division rate in the basal layer. Furthermore, as cells progress towards the upper layers, their size increases, which may influence the rest length under delamination conditions.
